# Application of Exogenous Phytohormones at Silking Stage Improve Grain Quality under Post-Silking Drought Stress in Waxy Maize

**DOI:** 10.3390/plants10010048

**Published:** 2020-12-28

**Authors:** Longfei Wang, Yini Yan, Weiping Lu, Dalei Lu

**Affiliations:** 1 Jiangsu Key Laboratory of Crop Genetics and Physiology, Jiangsu Key Laboratory of Crop Cultivation and Physiology, Jiangsu Co-Innovation Center for Modern Production Technology of Grain Crops, Yangzhou 225009, China; 18336070453@163.com (L.W.); trip741@163.com (Y.Y.); wplu@yzu.edu.cn (W.L.); 2 Joint International Research Laboratory of Agriculture and Agri-Product Safety, Ministry of Education of China, Yangzhou 225009, China

**Keywords:** grain weight, pasting viscosity, plant growth regulator, retrogradation, starch granule size, water deficit, waxy maize

## Abstract

The application of exogenous plant growth regulator can improve plant resistance to drought stress. The effects of application of exogenous cytokinin (CTK), brassinolide (BR), or gibberellic acid (GA) at the silking time on the grain quality of two waxy maize hybrids under drought stress at grain formation stage were studied. Grain weight of both hybrids was unaffected by exogenous phytohormones under control conditions but increased under drought conditions with the application of BR. The grain starch content in response to drought varied with hybrid and phytohormone. Starch granule size and protein content in grains were increased by drought under all conditions, but various phytohormones exerted different forms of influence. The starch λ_max_ in Yunuo7 was unaffected by single or interaction of phytohormones and water deficit, λ_max_ in Jingkenuo2000 with BR was unaffected but with CTK or GA increased by drought. Relative crystallinity was reduced by drought without the application of phytohormone, but with phytohormones in response to drought it was different. Flour peak viscosity was reduced by drought. The value was increased with BR spraying under control and drought conditions. Retrogradation percentage under drought conditions was unaffected by exogenous phytohormones in Jingkenuo2000. In Yunuo7, retrogradation percentage was unaffected by BR but reduced by CTK and GA. In conclusion, spraying phytohormones at the silking stage can affect grain weight and starch quality, grains with a sticky taste can be improved by applying BR, and grains with low retrograde tendency can be produced by applying CTK.

## 1. Introduction

Maize (*Zea mays* L.) plays an important role in global food security as it is a staple food and a feed crop for millions of people [1]. Rainfall mainly contributes to maize survival; unpredictable precipitation often induces drought or waterlogging, which decreases grain yield and quality [2]. Among the different abiotic stresses, drought is the vital adversity that affects maize growth and development [3]. Drought-prone maize regions will expand in the future and increase the frequencies of yield instability and food supply insecurity at local and even global levels [4].

Starch is the primary component (65–70%) of grains, followed by protein. In most cases, starch content decreases, and protein content increases after plants suffer post-silking drought stress [5,6,7]. Grain starch is gradually deposited after pollination and endosperm cell differentiation, and its composition and physicochemical properties are affected when plants are subjected to drought at this stage. Post-silking water deficit reduces the number of endosperm cells and starch granules [8], decreases starch granule size, and ultimately restricts total starch and amylopectin accumulation [9,10,11]. The small sizes of starch granules under drought conditions can improve starch pasting viscosity [12]. However, post-silking short-time water deficit increases pasting viscosity by enlarging starch granules [13], and irrigation can reduce the starch breakdown rate and setback viscosity [14]. Xia et al. [15] reported that water deficit reduces the numbers and volumes of starch granules and thereby lowers peak and trough viscosities. Zhang et al. [16] reported that moderate drought enlarges the size of starch granules and increases the proportion of long chains, which induces high gelatinization temperatures and retrogradation percentage, but severe drought exerts an opposite influence. 

Maize is sensitive to drought at the flowering and grain developmental stages, and thus strategies for relieving stress and increasing yield sustainably are necessary [17]. The application of plant growth regulators is an important cropping practice for alleviating the negative influences of environmental stresses [18]. Brassinolide (BR) enhances maize drought tolerance by mediating physiological and metabolic activities, maintaining tissue water potential, and antioxidant enzyme activity under drought [19]. Gibberellic acid (GA) can improve maize drought tolerance by keeping membrane permeability and increasing the contents of chlorophyll, relative water, and macronutrients in leaves [20]. Application of exogenous 2-(3,4-Dichlorophenoxy) triethylamine [21], melatonin [22], glutathione [23], spermidine [24], and salicylic acid [25] are reported to improve the drought tolerance of maize. However, the influence of exogenous phytohormones on the maize grain quality remain poorly understood. The plant growth regulators (ethephon, chlormequat chloride, and trinexapac-ethyl) application reduce grain weight, but their effects on malting quality are generally inconsiderable [26]. Post-anthesis drought reduces the weight and starch content of grains and increases the percentage of large starch granules, and spermidine or aminoethoxyvinylglycine can increase the proportion of small starch granules [9]. Polyamines (spermidine or free spermine) mediate the effect of post-anthesis water shortage on starch deposition by regulating activities of starch biosynthetic enzymes, and improved polyamine levels under moderate drought levels increasing the volume of medium granules and the weight of inferior grains in wheat [27]. Paclobutrazol, GA, or 6-benzylaminopurine can improve the milling quality and nutrition traits of rice grains by increasing head rice rate and amylose content [28].

Starch is composed of nearly pure amylopectin in waxy maize, which endows its high viscosity, low retrograde, and easier to digest than normal maize starch, widely used in food or some non-food industries [29,30]. Drought is the main environmental stress in China. Previous studies reported that grain yield and starch quality are affected by post-silking water deficit, which restricts starch deposition, downregulates enzymatic activities related to starch synthesis, reduces the sizes of starch granules, decreases starch crystallinity, and shortens amylopectin chains [31,32,33]. However, little is known about the influence of exogenous phytohormones on maize grain quality under drought stress. Exogenous plant growth regulator is a simple and economical cropping practice for improving the maize drought tolerance [18]. We hypothesize that the foliar spraying of exogenous phytohormones can alleviate detrimental influences of drought on the grain starch quality of waxy maize. In the present study, three widely used phytohormones, namely, cytokinin (CTK), BR, and GA were sprayed at the silking stage, and their effects on grain quality of plants subjected to water deficit after pollination (lasting 15 days) were studied. These results offer a choice for using exogenous phytohormones in cultivating waxy maize under post-silking drought conditions. 

## 2. Results and Discussion

### 2.1. Grain Weight

**Figure 1:**
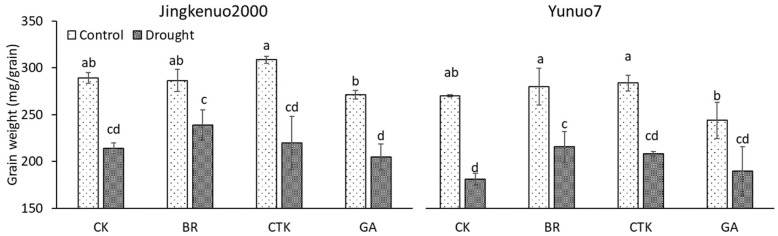
The grain weight with exogenous hormones under control and drought conditions in waxy maize. CK, water; CTK, cytokinin; BR, brassinolide; GA, gibberellic acid. Bars mean SE (n = 3). Different letters above the bars in each hybrid are significantly different at 5% probability level.

The grain weights of JKN2000 and YN7 significantly decreased by 26.0% and 33.0% under drought stress (Figure 1). Similar results were observed in fresh waxy maize [32], normal maize [34], barley [35], and wheat [9]. The decrease may be due to downregulated enzymatic activities related to starch synthesis and decreased IAA content [31]. Grain weight was unaffected by exogenous phytohormones under control conditions in both hybrids. Under drought conditions, grain weight in JKN2000 was also unaffected by phytohormones, the value in YN7 was increased only by exogenous BR and unaffected by CTK and GA. The foliar spraying of uniconazole with ethephon at the 12-leaf stage and 10 days after silking increased the contents of abscisic acid (ABA) and zeatin (Z) + zeatin riboside (ZR) but reduced the content of GA in grain, thereby increasing grain weight [36]. A study on rice observed that application of exogenous CTK (6-benzylaminopurine) at the late booting stage increases grain weight [37], but another study showed that grain weight in response to CTK (kinetin) under control and salt stresses varied among the tested hybrids [38]. A study on wheat showed that grain weight is unaffected by CTK (ZR) but is decreased by ABA [39].

### 2.2. Starch Content 

**Figure 2:**
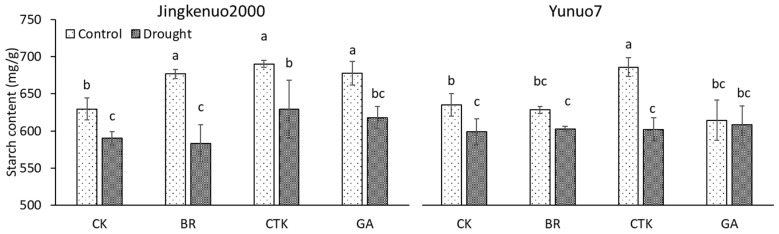
The grain starch content with exogenous hormones under control and drought conditions in waxy maize. CK, water; CTK, cytokinin; BR, brassinolide; GA, gibberellic acid. Bars mean SE (n = 3). Different letters above the bars in each hybrid are significantly different at 5% probability level.

Grain starch content was affected by the interaction between water deficit and plant growth regulator. In JKN2000, starch content was decreased by drought with or without phytohormones, especially with BR. In YN7, starch content with GA or BR was unaffected by drought, but the value with CTK or water decreased (Figure 2). Drought reduced grain starch content [5,6,7], owing to down-regulated enzymatic activities related to starch biosynthesis [31,40]. The decreased starch content under drought condition without phytohormones may be due to enlarged starch granules, which cannot compensated the reduced numbers of starch granules [11]. The starch content in normal maize grain was unaffected by the application of ethephon and diethyl aminoethyl hexanoate [41]. 

Under control condition, grain starch content in JKN2000 was significantly increased by exogenous phytohormones, but the increase was similar among the three phytohormones. Grain starch content in YN7 was unaffected by BR and GA but increased by CTK. Grain starch content can be increased by CTK (kinetin) [38]. Grain starch content was increased by spermidine and reduced by ethephon under both control and water deficit conditions [9]. Under water deficit condition, grain starch content in YN7 was similar among different treatments, the value in JKN2000 was unaffected by BR and GA but increased by CTK. 

### 2.3. Protein Content

**Figure 3:**
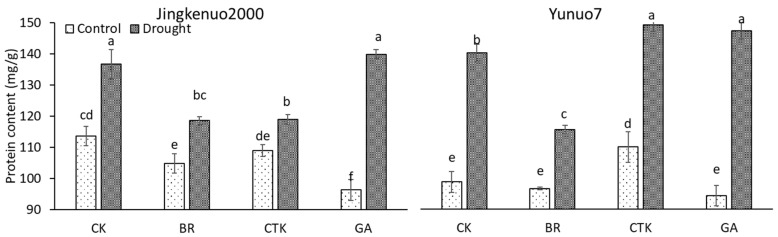
The grain protein content with exogenous hormones under control and drought conditions in waxy maize. CK, water; CTK, cytokinin; BR, brassinolide; GA, gibberellic acid. Bars mean SE (n = 3). Different letters above the bars in each hybrid are significantly different at 5% probability level.

Protein content in grain was increased by drought in both hybrids with or without plant growth regulators (Figure 3). This was similar to the result on wheat, which reported that drought enhances the sizes and relative areas of protein bodies in dorsal and abdominal endosperms [42]. In JKN2000, protein content under control condition was decreased with exogenous phytohormones, the value under drought condition was unaffected by GA and decreased by BR and CTK. In YN7, protein content under control condition was increased by CTK and unaffected by BR and GA, the value under drought condition was decreased by BR and increased by CTK and GA. A study on wheat found that the protein content in grain was unchanged by the application of CTK (ZR) but was increased by ABA [15]. McMillan et al. [26] reported that application of ethephon, chlormequat chloride, and trinexapac-ethyl did not affect the grain protein content in barley. 

### 2.4. Starch Granule Size (SGS)

**Figure 4:**
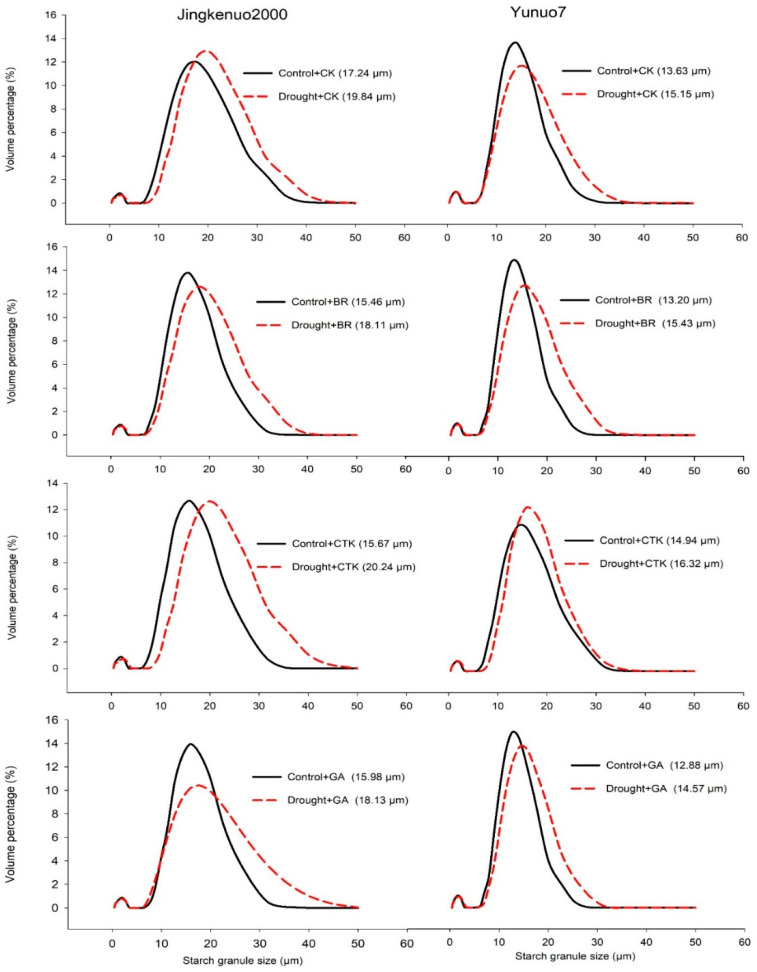
The size distribution of grain starch granules with exogenous hormones under control and drought conditions in waxy maize. CK, water; CTK, cytokinin; BR, brassinolide; GA, gibberellic acid. Data in the bracket are the average starch granule size.

Drought stress greatly enlarged the average SGS with or without spraying exogenous phytohormones in both hybrids (Figure 4). Drought at whole grain developmental stage decreased the SGS [32]. A study on wheat found that the SGS in response to post-anthesis drought was different among various hybrids [43]. The development of starch granules in grain divided into two stages, increase the numbers of starch granules at the first stage, following by the enlargement of starch granules [44]. The enlarged size of starch granules under drought stress in present study may be caused by re-watering induced photoassimilates transferred to the surviving starch granules and enlarged its size, as drought at the grain formation stage reduced the number of endosperm cells and starch granules [8]. Short-term water deficit increases starch granule size in wheat [13]. The SGS in JKN2000 was significantly larger than that in YN7. 

In YN7, the SGS under control condition was decreased by BR and GA but enlarged by CTK, and the SGS under drought condition was enlarged by BR and CTK but reduced by GA. In JKN2000, the SGS under control condition was decreased with all the exogenous phytohormones; under drought condition, it was reduced by BR and GA but enlarged by CTK. However, a research on wheat showed that the percentage of large starch granules was reduced by ABA but increased by GA_3_ [45]. Studies on rice [37] and wheat [46] demonstrated that the application of exogenous CTK increases endosperm cells and promotes nutrients to developing endosperms. GA has an important role in early grain development [47]. The distributions of starch granules in response to exogenous phytohormones varies among hybrid and water status [9]. To understand the effect of the application of exogenous phytohormones on grain development, accurate information on the enzymatic activities and contents of phytohormones related to starch synthesis and starch granule development need further consideration. 

### 2.5. X-ray Diffraction (XRD) Pattern

**Figure 5:**
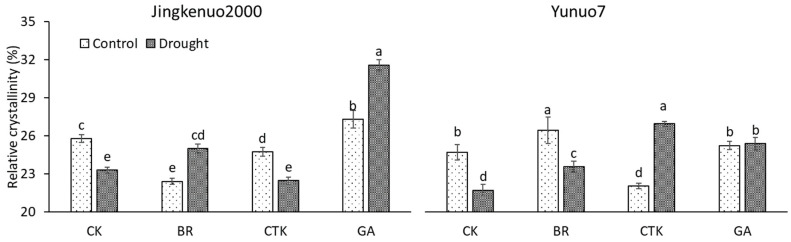
The flour relative crystallinity with exogenous hormones under control and drought conditions in waxy maize. CK, water; CTK, cytokinin; BR, brassinolide; GA, gibberellic acid. Bars mean SE (n = 3). Different letters above the bars in each hybrid are significantly different at 5% probability level.

The XRD profiles of all the samples present a typical A-type pattern (data not shown), but the relative crystallinity (RC) was affected by drought and phytohormone (Figure 5). In JKN2000, the RC was increased by drought with BR and GA and decreased with CTK and water, respectively. The RC under the control condition was increased by GA and decreased by BR and CTK, the value under drought condition was not infected by CTK but improved by BR and GA. In YN7, the RC was decreased by drought with water and BR, increased with CTK, and unaffected with GA. The RC under the control condition was increased by BR, decreased by CTK, and unaffected by GA; it under drought condition was increased with all the phytohormones. The decreased RC under drought conditions similar to the reports on fresh waxy maize [32], *Trimezia juncifolia* [48] and wheat [15]. Changes in RC is a part of strategy to adapt to different growth environments. Considering the effects of different exogenous phytohormones, selecting a suitable plant growth regulator is a feasible cropping practice based on utilizations.

### 2.6. Iodine Staining

**Figure 6:**
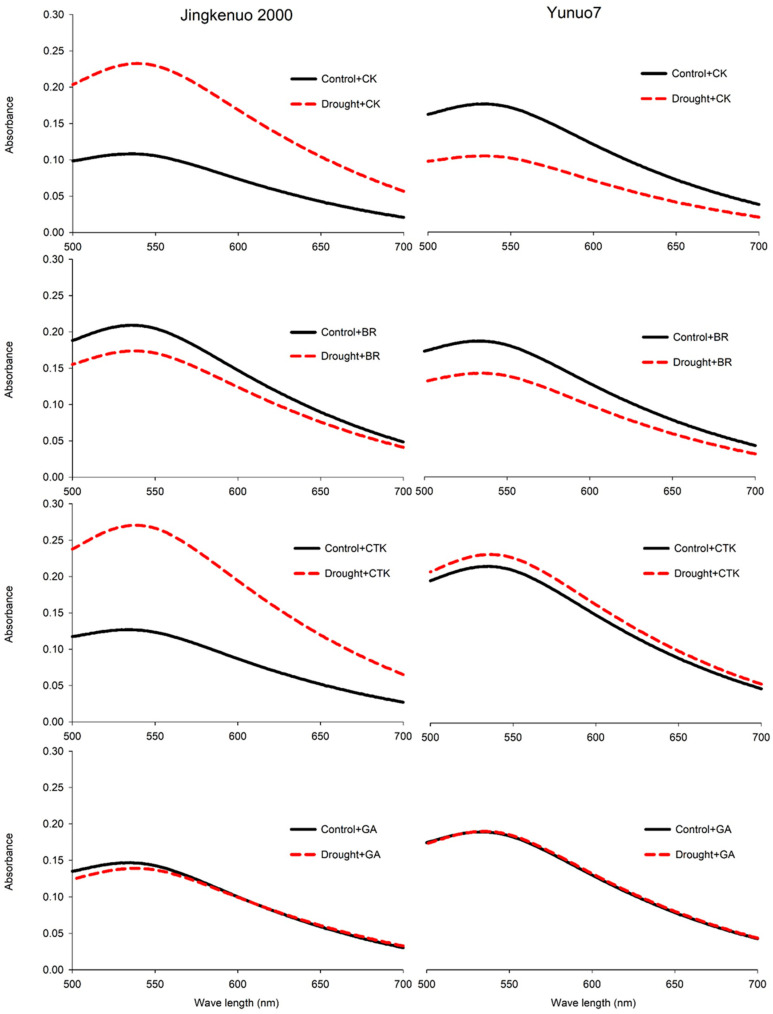
The starch wavelength distributions with exogenous hormones under control and drought conditions in waxy maize. CK, water; CTK, cytokinin; BR, brassinolide; GA, gibberellic acid.

Iodine can form a helical complex with amylopectin chains, and a high λ_max_ indicates that starch has a high proportion of long chains [49]. The λ_max_ of starch from waxy rice and maize was approximately 530–540 nm, present typical waxy character [32,49]. The starch λ_max_ in JKN2000 ranged from 534.7 nm to 535.3 nm and from 535.3 to 540.3 nm under control and drought conditions, respectively (Figure 6). The λ_max_ with BR was unaffected by drought, but it increased under other treatments. The starch λ_max_ in YN7 ranged from 533.3 nm to 535.7 nm, and it was unaffected by phytohormone and water deficit. The starch λ_max_ in this hybrid was unaffected by drought during total grain developmental stage [32]. The starch λ_max_ was positively correlated to the SGS, indicating that a large SGS has a high proportion of long chains. Those results are consistent with the earlier report [32].

### 2.7. Pasting Property

Grain pasting characteristics were affected by drought, phytohormone, and hybrid (Table 1). YN7 had a higher pasting characteristic than JKN2000 in general. Drought reduced the peak (PV) and breakdown (BD) viscosities and increased pasting temperature (*P*_temp_) in both hybrids with or without exogenous phytohormones. In JKN2000, the trough (TV) and final (FV) viscosities were increased by drought only with GA and unaffected by drought in the other three treatments; setback (SB) viscosity was increased by drought under all treatments. In YN7, the TV and FV with CTK were decreased but with GA and BR were increased by drought. The SB with water and CTK was increased but with BR and GA was decreased by drought. Our early study observed that the PV and BD of flour and starch in waxy maize were decreased by post-silking water deficit [32,33]. However, studies on wheat reported that starch pasting viscosities were improved by post-anthesis drought though the changes of starch granule size were discordant [12,13,14,47]. In this work, the low pasting viscosities may be caused by large granule size and high proportion of long chains in starch, as those viscosities were negatively correlated to SGS and λ_max_. In addition, BD was negatively correlated to protein content, indicating that proteins coated on starch granules restricted the starch granules broken during heating [50].

Under control condition, BR increased the pasting characteristics in both hybrids. The pasting characteristics in JKN2000 were unaffected by application of CTK, this phytohormone did not affect PV and SB, decrease BD, and increase TV, FV, and *P*_temp_ in YN7. The application of GA increased PV and BD and decreased *P*_temp_ but did not affect TV and FV in JKN2000, it did not affect all the pasting parameters in YN7. Under drought condition, SB and *P*_temp_ were decreased by the application of phytohormones, PV, TV, BD, and FV were increased by BR and GA but unaffected by CTK in JKN2000. In YN7, the parameters were unaffected by CTK, but PV, TV, BD, FV were increased and SB and *P*_temp_ were decreased with spraying BR and GA. Study on rice observed that the application of exogenous hormones under ambient temperature unaffected the starch pasting viscosities but it under heat stress significantly improved the viscosities, alleviate the negative influence of high temperature [51].

### 2.8. Thermal Property

Gelatinization properties were affected by drought and exogenous phytohormones (Table 2). In JKN2000, Δ*H*_gel_ was decreased by drought under different treatments. The Δ*H*_gel_ under control condition was lowest with GA and similar among the other three treatments, and the difference disappeared under drought condition. In YN7, the Δ*H*_gel_ was unaffected and decreased with exogenous phytohormones under control and water shortage conditions, respectively. The Δ*H*_gel_ was increased without phytohormones, with BR and CTK were unaffected, and with GA was decreased by drought. In JKN2000, the *T*_o_ with water, CTK, and GA increased, but the value with BR decreased by drought. The *T*_p_ with BR was unaffected by drought and increased in the other three treatments. The *T*_c_ with CTK was unaffected, with BR was decreased, but with GA and water was increased by drought. In YN7, the gelatinization temperatures increased by drought under all conditions. The Δ*H*_gel_ of flour or starch was unaffected by drought during whole grain filling stage, but gelatinization temperatures in response to water deficit were different among different waxy maize hybrids [32,33]. Zhang et al. [16] observed that moderate drought during grain filling increased starch gelatinization temperatures and reduced Δ*H*_gel_ in wheat, but severe drought produced a contrary influence. 

In YN7, *T*_o_ under control condition was increased by exogenous phytohormones, and *T*_p_ and *T*_c_ were unaffected by GA and increased by BR and CTK. Under drought condition, the gelatinization temperatures were unaffected by GA, decreased by BR, and increased by CTK, respectively. In JKN2000, the gelatinization temperatures under control condition were increased by exogenous phytohormones. Under drought conditions, the *T*_o_ and *T*_p_ were increased by GA and decreased by BR and CTK, *T*_c_ was increased by GA, decreased by BR, and unaffected by CTK. The gelatinization temperatures were positively correlated with protein content (Table 3), demonstrating that flour under drought conditions produced higher protein contents affected the gelatinization, similar to the influences on *P*_temp_. 

In JKN2000, the Δ*H*_ret_ with water and BR was decreased, but with CTK and GA was unaffected by drought. In comparison with control, the *%R* decreased without exogenous phytohormones, unaffected with BR, but increased with CTK and GA under drought condition. The Δ*H*_ret_ of YN7 under drought increased with BR and water, decreased with GA, and unaffected by CTK, the *%R* was decreased with CTK and GA and increased with BR and water. Our previous studies reported that Δ*H*_ret_ and *%R* were increased by post-silking water deficit [32,33]. Zhang et al. [16] found that the Δ*H*_ret_ and *%R* of wheat starch were increased and decreased by moderate and severe drought stresses, respectively. The discrepancy may due to the different drought tolerance, drought degree, and duration. The different responses to exogenous hormones provide a reference for the selection of hybrids and exogenous hormone based on utilization.

In YN7, the Δ*H*_ret_ under control condition was unaffected by BR and CTK but increased by GA, the value under drought condition was unaffected by BR but decreased by CTK and GA. The *%R* under control was increased by GA and decreased by BR and CTK, and *%R* under drought condition was unaffected by BR but was decreased by CTK and GA. In JKN2000, the Δ*H*_ret_ under both conditions decreased with exogenous phytohormones, the *%R* was reduced with exogenous phytohormones under control condition but the influence was disappeared under drought condition. The Δ*H*_ret_ and *%R* were negatively correlated to starch content, SGS and λ_max_, indicated that high starch content in grains, large granule size, and high proportion of long chains can reduce the retrogradation. For both hybrids under both water levels, the *%R* was lowest with CTK, indicated that the application of CTK could produce grains with low retrograde tendency. 

## 3. Materials and Methods

### 3.1. Experimental Design

A pot trial was conducted at the Experimental Farm of Yangzhou University in 2019. Two waxy maize hybrids, namely Jingkenuo2000 (JKN2000) and Yunuo7 (YN7), were used as materials. The pots, 38 cm in height and 43 cm in diameter, were loaded with 30 kg of sieved sandy loam soil from field. To each pot, 1.5 g of N, P_2_O_5_, and K_2_O was applied at the transplanting time, and 3.0 g of N was applied at the jointing stage.

Plants were grown in the field environment before the silking. The plants with similar appearance were sampled and manual pollinated on the same day, the exogenous phytohormones were sprayed on the plants at the afternoon (16:00–18:00). The treatments including water (CK), 0.1 g/L CTK [52], 0.025 g/L BR [53], or 0.02 g/L of GA [45], all solutions contains Tween 20 (0.05%) as the surfactant and the spraying volume was 100 mL/plant. The pots were moved to a mobile rain shelter for water treatments in the next day. Well-watered (75%‒80% of field moisture capacity) and drought (50%–55% of field moisture capacity), were imposed from silking. Soil moisture content was monitoring by a soil moisture sensor and evaporated water was supplied through a weighing method every morning. The period of drought stress lasted 15 days (grain formation stage) and then the stressed plants were re-watered normally until maturity (40 d after pollination). Each treatment included 20 pots.

### 3.2. Grain Weight

Three independent ears of each treatment were harvested at maturity. The kernels were stripped off ears, sun-dried and the weight (mg/grain) was weighed. After weighing, the grains (100 g, dry basis) were ground using a disintegrator (FW-100, Taisite, Tianjin, China), made to pass through a sieve with diameter 0.149 mm, and dried to constant weight at 60 °C. 

### 3.3. Grain Starch and Protein Contents Determination

Starch content in grain was measured by the anthrone-sulfuric acid method [54]. Nitrogen content in grain was determined following the procedure of AACC 46–10.01 [55], and protein content was calculated by measuring nitrogen content (protein content = nitrogen content × 6.25).

### 3.4. Starch Isolation

The starch in mature grains (100 g) was isolated using the method described in [29] after steeping in 500 mL of 1 g/L NaHSO_3_ solution at room temperature for 48 h.

### 3.5. Granule Size Distribution

Size distributions of starch granule were detected according to the protocol in [56]. 

### 3.6. Iodine Staining

The maximum absorption wavelength (λ_max_) of starch was measured according to the procedure in [56].

### 3.7. X-ray Diffraction Pattern

The X-ray diffraction (XRD) patterns of starch were obtained by an X-ray diffractometer (D8 Advance, Bruker-AXS, Germany), and the relative crystallinity (RC, %) was calculated according to the procedure in [56]. 

### 3.8. Pasting Properties

The pasting properties of flours (28 g total weight; 10% dry basis, *w*/*w*) were measured according to the procedure in [29]. 

### 3.9. Thermal Properties

The thermal properties of flours were determined with a differential scanning calorimetry (200 F3 Maia, NETZSCH, Bavaria, Germany) according to the procedure in [29]. The thermal transitions were defined as onset temperature (*T*_o_), peak gelatinization temperature (*T*_p_), conclusion temperature (*T*_c_), and gelatinization enthalpy (Δ*H*_gel_). Samples were stored at 4 °C for 7 days after thermal analysis for retrogradation investigations. Retrogradation enthalpy (Δ*H*_ret_) was automatically calculated, and retrogradation percentage (%*R*) was calculated as %*R* = 100 × Δ*H*_ret_ /Δ*H*_gel_.

### 3.10. Statistical Analysis

Data presented in the tables and figures were the mean value of three replications. ANOVA was used to determine the LSD at *p* < 0.05 level by a data processing system (version 7.05) [57].

## 4. Conclusions

The grain weight under normal condition was unaffected by spraying exogenous phytohormones at silking stage, but it under drought condition was increased by spraying BR in both hybrids. The grain starch and protein contents, starch granule sizes, λ_max_, and starch relative crystallinity were affected by the water deficit at 1–15 day after pollination, but exogenous phytohormones changed these parameters. The correlation results indicated that large size of starch granules, high proportion of long chains reduced the pasting viscosities, and Δ*H*_ret_ and *%R*. Starch content in grain was negatively correlated to *%R*. High protein content results in high gelatinization temperatures. Among the three widely used plant growth regulators, the application of BR could improve the grain with sticky taste, while the application of CTK could improve the grain with low retrograde tendency. Between the two hybrids, the high viscosities in YN7 and low *%R* in JKN2000 endows its advantages on different utilizations. 

## Figures and Tables

**Table 1:** The flour pasting properties with spraying exogenous phytohormones under post-silking control and drought conditions.

Hybrid	Soil Moisture	Exogenous Hormones	PV (cP)	TV (cP)	BD (cP)	FV (cP)	SB (cP)	*P*_temp_ (°C)
JKN2000	Control	CK	413 c	234 b	179 c	353 b	119 cd	76.8 c
		BR	701 a	308 a	389 a	434 a	123 bcd	74.9 d
		CTK	418 c	237 b	182 c	351 b	114 d	76.7 c
		GA	498 b	204 b	291 b	320 b	114 d	75.1 d
	Drought	CK	246 d	235 b	85 de	347 b	138 a	79.3 a
		BR	402 c	291 a	138 cd	421 a	131 abc	76.9 bc
		CTK	282 d	212 b	71 e	332 b	120 cd	77.0 bc
		GA	381 c	293 a	91 de	421 a	135 ab	78.3 ab
YN7	Control	CK	1516 b	731 ef	771 b	913 c	177 cd	76.7 cd
		BR	1650 a	811 c	839 a	1009 b	198 a	75.9 d
		CTK	1473 b	880 ab	593 c	1062 a	182 bc	78.3 b
		GA	1512 b	709 f	803 ab	883 c	174 cd	76.2 d
	Drought	CK	979 d	770 de	209 e	962 b	191 ab	79.4 a
		BR	1492 b	882 a	610 c	1063 a	181 bc	77.5 bc
		CTK	981 d	801 cd	179 e	999 b	198 a	79.7 a
		GA	1354 c	841 bc	513 d	1007 b	167 d	78.3 b
*F*-value								
Hybrid			6690.3 **	6865.8**	2145.3 **	6025.3 **	722.3 **	19.1 **
Soil moisture			492.0 **	15.6 **	1037.9 **	13.0 **	11.5 **	95.4 **
Hormone			128.1 **	26.7 **	166.7 **	24.0 **	4.7 **	16.5 **
Hybrid × Soil moisture			45.3 **	4.7 **	157.7 **	2.5	7.1 **	0.0
Hybrid × Hormone			8.1 **	11.5 **	34.6 **	10.6 **	6.5 **	5.2 **
Soil moisture × Hormone			16.8 **	24.8 **	6.0 **	15.9 **	4.1 *	4.5 *
Hybrid × Soil moisture × Hormone			28.9 **	5.0 **	43.0 **	2.4	4.0 *	1.3

JKN2000, Jingkenuo2000; YN7, Yunuo7; CK, control; BR, brassinolide; CTK, cytokinin; GA, gibberellic acid; PV, peak viscosity; TV, trough viscosity; BD, breakdown viscosity; FV, final viscosity; SB, setback viscosity; *P*_temp_, pasting temperature; cP, centipoise. Mean value in the same column within each hybrid followed by different letters are significantly different (*p* < 0.05). *, *p* < 0.05; **, *p* < 0.01.

**Table 2:** The flour thermal properties with spraying exogenous phytohormones under post-silking control and drought conditions.

Hybrid	Soil Moisture	Exogenous Hormones	Δ*H*_gel_ (J/g)	*T*_o_ (°C)	*T*_p_ (°C)	*T*_c_ (°C)	Δ*H*_ret_ (J/g)	%*R* (%)
JKN2000	Control	CK	9.2 a	68.7 e	73.9 e	83.1 c	3.6 a	39.1 a
		BR	9.3 a	69.5 cd	74.8 d	83.9 b	3.2 b	33.9 b
		CTK	9.0 a	69.4 d	74.7 d	84.1 b	2.1 e	23.2 d
		GA	8.1 b	69.9 c	75.4 c	84.4 b	2.1 de	26.3 c
	Drought	CK	7.3 c	70.8 b	76.1 b	84.4 b	2.5 c	34.6 b
		BR	7.0 c	68.8 e	74.7 d	82.4 d	2.3 de	32.4 b
		CTK	6.9 c	69.7 cd	75.5 c	84.3 b	2.2 de	32.3 b
		GA	7.1 c	72.0 a	77.1 a	85.7 a	2.3 cd	32.8 b
YN7	Control	CK	7.4 bc	68.1 f	73.4 f	81.5 d	3.2 b	42.9 d
		BR	7.8 ab	68.6 e	74.5 e	82.5 c	3.1 bc	39.5 e
		CTK	7.8 ab	69.6 d	75.0 d	82.4 c	3.0 bc	39.0 e
		GA	7.7 b	68.8 e	73.7 f	82.1 cd	4.0 a	51.8 a
	Drought	CK	8.2 a	71.4 b	76.9 ab	84.2 a	4.1 a	49.4 b
		BR	7.8 ab	70.1 c	76.1 c	83.4 b	3.8 a	48.4 b
		CTK	7.4 bc	71.8 a	77.2 a	84.6 a	2.8 c	37.1 f
		GA	7.1 c	71.1 b	76.5 b	84.8 a	3.3 b	46.0 c
*F*-value								
Hybrid			15.0 **	1.3	5.6 *	71.5 **	307.9 **	1332.4 **
Soil moisture			105.3 **	639.4 **	1052.2 **	159.9 **	7.2 *	39.7 **
Hormone			7.3 **	62.2 **	39.9 **	31.5 **	49.2 **	115.4 **
Hybrid × Soil moisture			101.3 **	101.3 **	151.1 **	85.1 **	34.1 **	0.4
Hybrid × Hormone			0.3	48.7 **	65.5 **	7.9 **	14.8 **	46.4 **
Soil moisture × Hormone			2.9	64.3 **	69.0 **	33.0 **	0.9	6.6 **
Hybrid × Soil moisture × Hormone			7.2 **	11.8 **	1.3	1.6	54.9 **	89.9 **

JKN2000, Jingkenuo2000; YN7, Yunuo7; CK, control; BR, brassinolide; CTK, cytokinin; GA, gibberellic acid; Δ*H*_gel_, gelatinization enthalpy; *T*_o_, onset temperature; *T*_p_, peak gelatinization temperature; *T*_c_, conclusion temperature; Δ*H*_ret_, retrogradation enthalpy; %*R*, retrogradation percentage. Mean value in the same column within each hybrid followed by different letters are significantly different (*p* < 0.05). *, *p* < 0.05; **, *p* < 0.01.

**Table 3:** Correlation coefficients between various characteristics (n = 16).

	PC	SC	SGS	RC	λ_max_	Δ*H*_gel_	*T*_o_	*T*_p_	*T*_c_	Δ*H*_ret_	*%R*	PV	TV	BD	FV	SB
**SC**	−0.56 *															
**SGS**	0.43	−0.25														
**RC**	0.11	−0.08	0.02													
**λ** **_max_**	0.32	−0.32	0.83 **	0.05												
**Δ*H*_gel_**	0.39	−0.20	−0.26	0.01	−0.43											
***T*** **_o_**	−0.38	0.58 *	0.32	−0.21	0.34	−0.28										
***T*** **_p_**	0.86 **	−0.31	0.36	0.25	0.31	−0.34	0.97 **									
***T*** **_c_**	0.85 **	−0.36	0.51 *	0.18	0.46	−0.07	0.85 **	0.82 **								
**Δ*H*_ret_**	0.69 **	−0.06	−0.59 *	0.33	−0.52 *	0.25	−0.09	−0.13	−0.37							
***%R***	−0.04	−0.27	−0.52 *	−0.34	−0.36	−0.16	0.01	0.00	−0.35	0.91 **						
**PV**	0.10	−0.49 *	−0.85 **	−0.26	−0.60 *	−0.11	−0.22	−0.19	−0.54 *	0.65 **	0.73 **					
**TV**	−0.25	−0.03	−0.70 **	−0.15	−0.51 *	−0.22	0.08	0.12	−0.34	0.65 **	0.76 **	0.92 **				
**BD**	0.10	−0.21	−0.86 **	−0.18	−0.57 *	0.00	−0.49 *	−0.49 *	−0.67 **	0.54 *	0.58 *	0.92 **	0.70 **			
**FV**	−0.56 *	0.13	−0.70 **	−0.10	−0.52 *	−0.22	0.08	0.12	−0.34	0.65 **	0.76 **	0.92 **	1.00 **	0.70 **		
**SB**	0.10	−0.21	−0.60 *	−0.18	−0.43	−0.29	0.14	0.18	−0.31	0.60 *	0.73 **	0.84 **	0.95 **	0.61 **	0.95 **	
***P*** **_temp_**	0.86 **	−0.54 *	0.29	−0.06	0.34	−0.43	0.72 **	0.70 **	0.37	0.10	0.27	−0.01	0.33	−0.34	0.33	0.43

PC, protein content; SC, starch content; SGS, starch granule size; RC, relative crystallinity; λmax, maximum wavelength; PV, peak viscosity; TV, trough viscosity; BD, breakdown viscosity; FV, final viscosity; SB, setback viscosity; *P*_temp_, pasting temperature; Δ*H*_gel_, gelatinization enthalpy; *T*_o_, onset temperature; *T*_p_, peak gelatinization temperature; *T*_c_, conclusion temperature; Δ*H*_ret_, retrogradation enthalpy; %*R*, retrogradation percentage. * Correlation is significant (*p* < 0.05). ** Correlation is significant (*p* < 0.01).

## Data Availability

The data presented in this study is contained within the article.
